# The Role of Fine Needle Aspiration of Liver and Spleen in the Staging of Low-Grade Canine Cutaneous Mast Cell Tumor

**DOI:** 10.3390/vetsci9090473

**Published:** 2022-09-01

**Authors:** Valentina Rinaldi, Paolo Emidio Crisi, Massimo Vignoli, Alessio Pierini, Rossella Terragni, Emanuele Cabibbo, Andrea Boari, Riccardo Finotello

**Affiliations:** 1 Faculty of Veterinary Medicine, University of Teramo, 64100 Teramo, Italy; 2 Department of Veterinary Sciences, Veterinary Teaching Hospital “Mario Modenato”, University of Pisa, 56122 Pisa, Italy; 3 Clinica Veterinaria Pet Care, Animalia, via Marzabotto 1/2 M-N, 40133 Bologna, Italy; 4 Clinica Veterinaria Jenner, VetPartners, via Jenner 37, 43126 Parma, Italy; 5 Department of Small Animal Clinical Science, Institute of Infection, Veterinary and Ecological Science, University of Liverpool, Chester High Road, Neston CH64 7TE, UK

**Keywords:** mast cell tumor, FNA, dog, liver, spleen

## Abstract

**Simple Summary:**

Mast cell tumor is one of the most common cutaneous tumors in dogs, representing 16–21% of all cutaneous tumors. Clinical staging is a fundamental step in the patients’ assessment, and it is considered essential to further refine the prognosis and to establish an ad hoc therapeutic plan. The recommended staging work-up includes a basic laboratory database, fine needle aspiration of the expected tumor draining lymph node/s, and diagnostic imaging with fine needle aspiration of the liver and spleen. The aim of this retrospective study was to investigate the incidence, at presentation, of hepatic and splenic metastases in dogs affected by low-grade cutaneous mast cell tumor referred for further investigations (Patnaik grade I–II, Kiupel low-grade). Only 1 out of 136 dogs had the presence of visceral metastases at diagnosis, suggesting that the prevalence of visceral metastases in low-grade cutaneous mast cell tumor is extremely low and that cytology of visceral organs may not represent an essential step in the clinical staging work-up in these dogs.

**Abstract:**

Clinical staging is a fundamental step in the clinical assessment of canine cutaneous mast cell tumor (cMCT), and it is recommended to evaluate the tumor draining lymph node (eTDLN), perform diagnostic imaging, and fine needle aspiration (FNA) of the spleen and liver to determine the presence of metastatic disease, thereby refining the prognosis. The aim of this retrospective study was to evaluate the prevalence of splenic and hepatic involvement in newly diagnosed canine low-grade cMCT (Patnaik grade I–II, Kiupel low-grade). Medical records of dogs that underwent a clinical staging work-up and surgical excision for a low-grade cMCT between December 2019 and December 2021 were reviewed at five veterinary centers. Only dogs with a histological diagnosis of low-grade cMCT, FNA or histology of the eTDLN, FNA of the spleen and liver, and one year of follow up were included. One hundred and thirty-six dogs met the inclusion criteria. Only 1 out of 136 dogs (0.7%) had the presence of visceral metastases at diagnosis, suggesting that the prevalence of visceral metastases in low-grade cMCT is extremely low. The results of this study are consistent with previous literature and suggest that after a diagnosis of low-grade cMCT, cytology of visceral organs may not represent an essential step in the clinical staging work-up.

## 1. Introduction

Mast cell tumor (MCT) is one of the most common cutaneous tumors in dogs, representing 16–21% of all cutaneous tumors [1,2]. The Patnaik three-tier grading system has been the foundation for establishing a prognosis since 1984 [3], with numerous studies having proved its reliability in the clinical setting. Patnaik grade I (P-GI) cMCTs have an excellent long-term prognosis, while Patnaik grade III (P-GIII) are associated with a guarded to poor prognosis [4]. Conversely, predicting the behavior of Patnaik grade II (P-GII) cMCTs has been proven challenging: although many would have a good prognosis, 20–50% appear to be characterized by a more aggressive clinical behavior and are associated with a high risk of tumor-related deaths [4]. These issues led to the development of a two-tier grading system by Kiupel and colleagues in 2011 [5], dividing cMCT into low-grade (K-LG) and high-grade (K-HG). Kiupel’s grading system has been validated in multiple studies and has increased the concordance among pathologists, although a combination of Patnaik and Kiupel’s gradings system is currently preferred, as it decreases inter-observer variation and provides 96.8% inter-observer consistency [6]. Although histological grading represents the main prognostic factor [4,7,8], the clinical staging work-up is considered essential to further refine a prognosis and to establish a targeted therapeutic plan [4,9,10]. The recommended staging work-up includes a basic laboratory database, fine needle aspiration (FNA) of the expected tumor draining lymph node/s (eTDLN), and abdominal ultrasound (US) with FNA of the liver and spleen; thoracic radiography is also suggested. Bone marrow involvement is not commonly evaluated in cMCT, as it is affected in a minority of cases by cytopenias and/or visceral metastases being present [2]; metastases to the BM only occur in high-grade cMCT and are associated with a grave prognosis and even shorter survival times [11,12]. Different studies have highlighted the relevance of lymph node staging in mast cell tumors, suggesting that regional (RLN) and sentinel lymph nodes (SLN) differ in 25% [13] to 42% [14] of the tumors. The overall rate of nodal metastases in low-grade cMCTs is 18% [15] while the reported metastatic rate of high-grade tumors ranges from 55 to 96% [16]. Furthermore, it has been extensively demonstrated that the removal of metastatic eTDLN is associated with a better outcome in canine cMCT [17,18,19,20], supporting the relevance of assessing and monitoring these organs during follow-up. Thoracic radiographs rarely demonstrate metastasis and are mainly performed to exclude comorbidities or to assess intrathoracic lymphoid structures [2,21]. US is the most commonly used imaging method for evaluation of spleen and liver MCT metastases, with a sensitivity of 43–71% and 0–71%, respectively [22,23,24]; a specificity of 68% and 93% has been recently reported for the spleen and liver, respectively [24]. Hughes and colleagues [25] explored the use of computed tomography (CT) for MCT staging and concluded that evaluation of the liver showed no consistent pattern associated with metastasis and did not predict cytology results. Regardless of the histological grading, cMCTs are classified in four clinical stages according to the World Health Organization (WHO) [26]. The aim of this retrospective study was to investigate the incidence, at presentation, of hepatic and splenic metastases in dogs affected by cMCT. The authors hypothesized that the incidence would have been as low as previously suggested, therefore questioning the pivotal role of visceral organs’ cytology in the staging of low-grade cMCT.

## 2. Materials and Methods

### 2.1. Patient Selection and Staging

This retrospective investigation was conducted at five tertiary referral institutes: the Veterinary Teaching Hospital of the Faculty of Veterinary Medicine at University of Teramo (Italy), Department of Small Animal Clinical Science of Liverpool University (Neston, United Kingdom), Department of Veterinary Sciences of University of Pisa (Italy), the Veterinary Clinic Jenner, Vet Partners (Parma, Italy), and the Veterinary Clinic Pet Care, Animalia (Bologna, Italy). Medical records of dogs diagnosed with a de novo low-grade cMCT between December 2019 and December 2021 were reviewed; cMCT were defined as low-grade if had a histological diagnosis of P-GI, P-GII/K-LG, detailed according to both Patnaik and Kiupel’s grading systems [3,5]. To be included in the study, dogs had to satisfy the following criteria: (1) surgical excision of the primary tumor; (2) detailed record of a staging work-up, consisting of a physical examination, complete blood count, serum biochemistry, cytological or histological evaluation of the eTDLN, thoracic radiographs and abdominal US or CT scan, and FNA of the liver and spleen; and (3) at least 1-year telephone follow-up with the owner or referring veterinarian. Dogs that received neoadjuvant corticosteroid or neoadjuvant chemotherapy treatment were excluded from the study. Re-staging was repeated every three months, and this consisted of blood tests, evaluation of regional lymph nodes, and abdominal ultrasound. FNA of the liver and spleen was at the clinician’s discretion.

Percutaneous ultrasound-guided cytology of the liver and spleen was performed under sedation, and the organs were sampled regardless of their ultrasonographic appearance. The cytologic criteria used to define evidence of visceral metastasis included the presence of large numbers and/or clusters of well-differentiated mast cells or the presence of mast cells with an atypical morphology [27]. For the evaluation of eTDLN, the presence of metastatic disease was defined according to Krick’s system [28] for cytology and Weishaar’s grading system for histopathology [9]. Cytology and histology reports had to be validated by a board-certified specialist or by a professional with experience in the field. Clinical stage (0–IV) and substage (a–b) was classified according to the WHO staging system for canine MCT [26]. Signalment (breed, age, sex), cMCT anatomic location, size (>3 cm or ≤3 cm), and presence of ulceration were also recorded.

### 2.2. Statistics

Computer software was used to perform the analysis (GraphPad Prism version 6.01, GraphPad Software, La Jolla, CA, USA). Data were evaluated using a standard descriptive statistic and reported as the mean ± standard deviation (SD) or the median and range (minimum–maximum), based on their distribution. Normality was checked using the D’Agostino Pearson test. A Fischer’s exact test was used to evaluate the frequency of metastasis in dogs with Patnaik grade I and II. The level of statistical significance was set at *p* < 0.05.

## 3. Results

One hundred and thirty-six dogs fulfilled the inclusion criteria. There were 70 male (41 neutered) and 66 female (50 spayed) dogs with a median age of 84 months (minimum 12; maximum 168). The most represented breeds were crossbreed (*n* = 27; 19.85%), Labrador Retriever (*n* = 27; 19.85%), Boxer (*n* = 27; 19.85%), and other breeds (*n* = 55; 40.45%). Among the 136 dogs, the tumors were located on head and neck (*n* = 30; 22%), limbs (*n* = 50; 36.7%), trunk (*n* = 43; 31.6%), and inguinal region (*n* = 13; 9.5%). Of those, 6 out of 136 dogs presented with ulcerated lesions (3) or masses measuring > 3 cm (3). Among the LG-K cMCT, there were 13 cases with P-GI and 123 with P-GII; each dog was affected by one cMCT as there were no cases with multiple cMCT. All cMCT were removed with complete surgical margins and in 24 cases eTDLN was also excised. Based on the Weishaar’s grading system, the dissected lymph nodes were classified as HN0 in 6 cases, HN1 in 4 cases, HN2 in 12 cases, and HN3 in 2 cases [9]. In the remaining 112 dogs, where lymphadenectomy was not performed, eTDLN metastasis was described via cytology in 4 cases (probable and certain metastases) [28]. One dog underwent total body CT, while in 135 the staging was performed by thoracic radiographs and abdominal ultrasonography. Thoracic evaluation did not reveal lymphadenopathy or pulmonary abnormalities in any of the cases. Abdominal ultrasound showed no presence of overt ultrasound changes in any of the 136 dogs, including the dog with visceral metastases. Only one dog was positive to metastases (0.7%) in both the spleen and liver cytological exams. Among the 13 P-GI, only one dog had an eTDLN positive for metastasis (HN2) and none of the P-GI had evidence of distant visceral metastases. Among the P-GII dogs, 18 had metastases to the eTDLN (2 HN3, 11 HN2, and 4 by cytologic exam), while 1 dog had both liver and spleen metastasis, with eTDLN negative by histologic exam. No differences were observed in the occurrence of metastases between dogs with P-GI and P-GII (*p* = 1.000). One hundred and seventeen dogs were classified as WHO stage I, 18 as WHO stage II, and 1 as WHO stage IV, with both evidence of cMCT metastases to the liver and spleen. At the time of referral, all dogs were classified as WHO substage a. Data are summarized in Table 1.

Twenty-seven dogs in WHO clinical stage I–II were treated with antineoplastic treatments as follows: 27 dogs received dose-intense chemotherapy (vinblastine 2 mg/m^2^ +/− dose escalation), one dog received inhibitor tyrosine kinase (TKI) (Masitinib), and 2 dogs received vinblastine and TKI (Toceranib). The only patient in WHO stage IV received a standard vinblastine and prednisolone 12 weeks protocol; cytology of the liver and spleen was still positive for metastatic disease, three months after completing treatment. For this reason, the dog remains under treatment of Toceranib (2.7 mg/kg every Monday, Wednesday, and Friday).

The median follow-up time was 771 days (370–1900 days); 135 were reported to be alive and with no documented evidence of metastatic disease at the time of the writing. The patient in clinical stage IV remained alive (i.e., 370+) but was still positive for splenic and hepatic MCT involvement.

## 4. Discussion

The aim of this study was to mainly document the incidence of visceral metastases in LG-K cMCT, and to therefore discuss the benefit of liver and spleen cytology during initial and subsequent staging work-up in these patients. In this study, the most represented breeds were crossbreed dogs, followed by Labrador Retriever and Boxer dogs, which is in line with what previously has been reported [4,29]. The remain signalment features were also similar to what has been described in the literature [4], supporting that this population was representative of a common cMCT population. Concerning the rate of lymph node metastases, after dividing the population of LG-K into P-G1 and P-G2, we did not find a different risk among the two groups, which is in line with what previously has been reported [30,31]; however, this may also represent a type 2 error due to the small number of P-G1 dogs. Notably, the eTDLN was not involved in the dog with stage IV disease, differently from Warland et al. [21], where all dogs with distant metastases had also a positive lymph node. It is possible to hypothesize that some MCTs can metastasize to secondary sites alone via the blood vessels, bypassing the LN. Alternatively, it can be argued that the assessed LN was the RLN, which may not necessarily be the SLN [13,14]. Given that SLNs were not necessarily evaluated in the present study, this feature may represent a limit and some dogs may have been inadvertently classified as stage I. Furthermore, a recent paper reported a 31% false-negative result in RLN cytology when compared to histology [32]. Histologic exam remains the gold standard for a conclusive diagnosis [33] and lymph node dissection has been recommended also for non-palpable or normal-sized regional lymph nodes [34]. Interestingly, despite the presence of distal metastases, the only dog in this study who had stage IV disease was still alive at the time of writing, 370 days post diagnosis, which would be unexpected when referring to the study by Pizzoni et al. [35], where dogs with stage IV disease had an overall median survival time of 110 days. In our study, the percentage of visceral metastases was as low as 0.7%, which is similar to the 2% reported by Stefanello et al. [15] in a study conducted on a cohort of 295 dogs affected by K-LG cMCT. This is instead lower than the 8% reported by Warland et al. [21] in 186 dogs with P-GI and P-GII tumors; however, in that cohort the Kiupel grading was not performed and it should be expected for K-HG to have been included in the study and to possibly account for the discrepancy [21]. K-HG do in fact carry a much higher risk of distant metastatic disease [15]. In an abdominal ultrasound, the absence of overt ultrasound changes in dogs with visceral metastases has already been proven [23]. In our series, in addition to the low percentage of visceral metastases, there was no evidence of pulmonary involvement and/or hematological abnormalities. Although an initial comprehensive staging work-up is recommended in a cancer patient to also detect comorbidities, some investigations are less relevant for the purpose of specifically investigating the true extension of the neoplastic process. To the best of the authors’ knowledge, the percentage of pulmonary involvement is unknown [2,21]. In a recent consensus proposal, it has been suggested that thorough staging procedures, such as abdominal imaging with FNA of the liver and spleen, thoracic radiographs, and BM aspiration, should be reserved for patients with a positive eTDLN or with high risk of developing metastases [8], and our findings seem to further support such statements. Our study presents several limitations inherent to its retrospective and multicentric nature. For instance, there was lack of consistency in repeating staging work-up, therefore we cannot be entirely sure that distant metastases have not arisen during follow-up; however, the fact that all patients were alive at the time the manuscript has been written, would make this less likely. Moreover, several relevant information was not available, such as the Ki-67 expression, AgNOR scoring, KIT pattern, and the status of c-kit mutations; it is therefore possible that within our population, we have accidentally selected a particularly benign subset of cMCT. Furthermore, the relatively short follow-up has not allowed the authors to exclude that metastasis may occur in the long term.

## 5. Conclusions

In conclusion, in this group of study, the incidence of visceral metastases at presentation was particularly low (0.7%), suggesting that cytological evaluation of the liver and spleen does not represent an essential part of the staging work-up in dogs with K-LG MCT. Moreover, we have once again reported the absence of cMCT-related abnormalities in the thoracic radiographs of such populations.

## Figures and Tables

**Table 1:** Tumor characteristics and staging of 136 dogs affected by mast cell tumor.

Breed	Mix		Labrador	Boxer	Other Breeds	
	(*n* = 27), 19.85%		(*n* = 27), 19.85%	(*n* = 27), 19.85%	(*n* = 55), 40.45%	
Age	<3 y		3–8 y		>8 y	
	(*n* = 5), 3.67%		(*n* = 81), 59.56%		(*n* = 50), 36.77%	
Sex	Male		Female	Neutered Male	Spayed Female	
	(*n* = 29), 21.32%		(*n* = 16), 11.6%	(*n* = 41), 30.15%	(*n* = 50), 36.77%	
Anatomic Regional	Head and Neck		Limbs	Trunk	Inguinal Region	
	(*n* = 30), 22%		(*n* = 50), 36.80%	(*n* = 43), 31.70%	(*n* = 13), 9.50%	
WHO Stage	I		II	III	IV	
	(*n* = 117), 86%		(*n* = 18), 13.30%	(*n* = 0), 0	(*n* = 1), 0.70%	
eTDLN	HN0	HN1	HN2	HN3	Cytologic+	Cytologic−
	(*n* = 6), 4.40%	(*n* = 4), 2.90%	(*n* = 11), 8.85%	(*n* = 2), 1.40%	(*n* = 4), 2.90%	(*n* = 132), 79.45%
Ulcer	Yes				No	
	(*n* = 3), 2.20%				(*n* = 133), 97.87%	
Size	<3 cm				>3 cm	
	(*n* = 133), 97.80%				(*n* = 3), 2.20%	
Patnaik Grade	I		II		III	
	(*n* = 13), 9.55%		(*n* = 123), 90.45%		(*n* = 0), 0%	
Kiupel Grade	Low				High	
	(*n* = 136), 100%				(*n* = 0), 0%	

Abbreviation: WHO: World Health Organization; eTDLN: expected tumor draining lymph node; HN0: non metastatic; HN1: premetastatic; HN2: early metastasis; HN3: overt metastasis (Weishaar et al., 2014); cytologic exam +: positive for metastasis at cytological exam (Krick et al., 2009); cytologic exam −: negative for metastasis at cytological exam (Krick et al., 2009).

## Data Availability

The data that support the findings of this study are available upon request.
